# Dendritic cell function and pathogen-specific T cell immunity are inhibited in mice administered levonorgestrel prior to intranasal *Chlamydia trachomatis* infection

**DOI:** 10.1038/srep37723

**Published:** 2016-11-28

**Authors:** Nirk E. Quispe Calla, Rodolfo D. Vicetti Miguel, Ao Mei, Shumin Fan, Jocelyn R. Gilmore, Thomas L. Cherpes

**Affiliations:** 1 Department of Microbial infection & Immunity, The Ohio State University College of Medicine, Columbus, OH, 43210, USA; 2 Department of Obstetrics & Gynecology The Ohio State University College of Medicine, Columbus, OH, 43210, USA

## Abstract

The growing popularity of levonorgestrel (LNG)-releasing intra-uterine systems for long-acting reversible contraception provides strong impetus to define immunomodulatory properties of this exogenous progestin. In initial *in vitro* studies herein, we found LNG significantly impaired activation of human dendritic cell (DCs) and their capacity to promote allogeneic T cell proliferation. In follow-up studies in a murine model of intranasal *Chlamydia trachomatis* infection, we analogously found that LNG treatment prior to infection dramatically reduced CD40 expression in DCs isolated from draining lymph nodes at 2 days post infection (dpi). At 12 dpi, we also detected significantly fewer CD4^+^ and CD8^+^ T cells in the lungs of LNG-treated mice. This inhibition of DC activation and T cell expansion in LNG-treated mice also delayed chlamydial clearance and the resolution of pulmonary inflammation. Conversely, administering agonist anti-CD40 monoclonal antibody to LNG-treated mice at 1 dpi restored lung T cell numbers and chlamydial burden at 12 dpi to levels seen in infected controls. Together, these studies reveal that LNG suppresses DC activation and function, and inhibits formation of pathogen-specific T cell immunity. They also highlight the need for studies that define *in vivo* effects of LNG use on human host response to microbial pathogens.

Intra-uterine systems (IUSs) have become a popular choice for long-acting reversible contraception (LARC) worldwide^1^. While especially popular in Asia^2^, IUS use among contraceptors in the U.S. increased from 2.0% in 2002 to 10.3% in 2012 3. Among the 3 IUSs now approved in the U.S. for LARC, 2 release the exogenous progestin levonorgestrel (LNG). Expressly, Skyla^®^ (13.5 mg LNG) and Mirena^®^ (52 mg LNG) are approved for 3 and 5 years use, respectively^4^,^5^. Based on their effectiveness at preventing unintended pregnancy, The American College of Obstetricians and Gynecologists and The American Academy of Pediatrics identified LNG-IUSs as top-tier LARC choices for women and adolescents^6^,^7^. Despite the increasingly widespread LNG-IUS utilization, only a limited number of laboratory animal and clinical studies have explored the effects of LNG on mechanisms of anti-pathogen host defense.

As examples, LNG-treated mice showed greater genital mucosal permeability and susceptibility to virus infection^8^, while multiple clinical studies identified IUSs users as most likely to develop pelvic inflammatory disease (PID) during the first 3 weeks after IUS insertion^9^,^10^,^11^. Conversely, the incidence of acquiring sexually transmitted infection among women using LNG-IUS vs. no hormonal contraceptive is unexplored by prospective longitudinal study. Also underexplored are the *in vivo* effects of LNG on pathogen clearance. One retrospective study did observe reduced genital clearance of high-risk human papillomavirus (HPV) in women using LNG-IUS^12^, while *Chlamydia trachomatis* clearance was delayed in baboons infected subsequent to human-use LNG-IUS insertion^13^. Notably however, the immunomodulatory properties of LNG responsible for these experimental and clinical observations have not been defined.

Our laboratory previously reported dendritic cell (DC) activation and development of virus-specific immunological memory were inhibited in mice administered medroxyprogesterone acetate (MPA) prior to corneal infection with herpes simplex virus type 1 (HSV-1)^14^. MPA is the active component of the progestin-only, injectable hormonal contraceptive Depo-Provera. In the current study, we used *in vitro* assays with human DCs and a murine model of intranasal *C. trachomatis* infection to similarly explore the influence of LNG on early anti-pathogen immune responses. This animal model of respiratory infection represents an important complement to the mouse urogenital model, and can delineate chlamydial pathogenesis and host-chlamydia interactions, screen antimicrobials for anti-chlamydial activity, and gage efficacy of candidate *Chlamydia* vaccines^15^.

## Results

### LNG suppressed human DC activation and function

**Figure 1:**
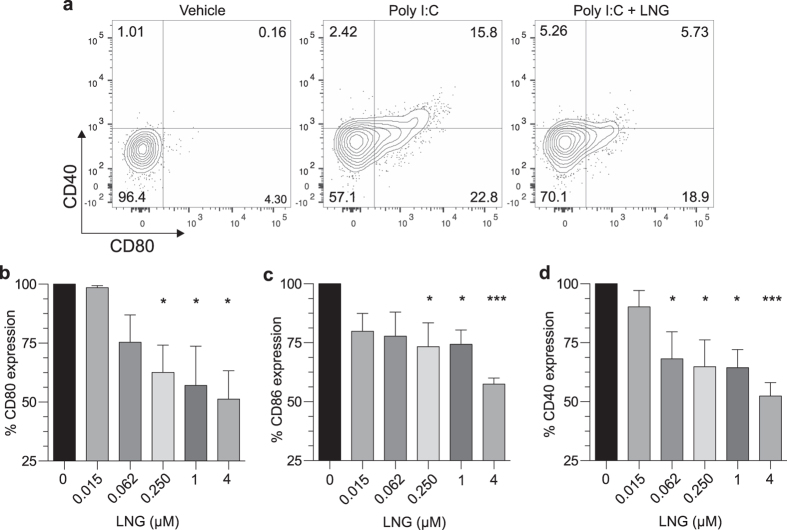
LNG inhibits human DC activation. Negatively selected human DCs were incubated for 24 h with indicated LNG concentrations or vehicle alone, then incubated for 24 h with poly I:C (1.5 μg/mL). DCs were stained with a live/dead near-IR dye and a panel of fluorescently-tagged mAbs to identify viable DC populations by flow cytometry (described in Materials and Methods). (**a**) Representative contour plots of CD40 and CD80 expression by untreated or LNG (4 μM)-treated mDCs stimulated with poly I:C; quadrant numbers denote percent expression. (**b–d**) mDC expression of (**b**) CD80, (**c**) CD86, and (**d**) CD40 after poly I:C stimulation. Data from 8 independent experiments with results normalized (i.e., by designating vehicle-only cultures as 100% activation) as detailed in Materials and Methods (bars denote means ± SD). Statistical analyses performed using 1-way ANOVA with Dunnett’s multiple comparisons test, *p < 0.05; ***p < 0.001.

In prior studies, MPA modulated *in vivo* immune responses of mice to HSV-1 infection^14^, and inhibited human DC activation and function *in vitro*^16^. As MPA binds the glucocorticoid (GR) and progesterone receptors (PR)^17^, these findings may have been mediated by interactions of MPA with either receptor. While LNG more selectively binds the PR^18^, MPA and LNG did similarly increase mouse genital mucosal permeability and susceptibility to HSV-2 infection^8^. Therefore, we began the current study by positing LNG analogously inhibits DC activation and function. To first test this hypothesis, we isolated human primary DCs from the peripheral blood of 8 individuals by negative immunomagnetic selection. Selected cells were incubated for 24 h with vehicle alone or concentrations of LNG that ranged between 0.015 μM–4 μM. Cells were incubated an additional 24 h after adding the Toll-like receptor 3 (TLR3) agonist polyinosinic: polycytidylic (poly I:C) (1.5 μg/mL). Interrogation of these cells in flow cytometry-based studies revealed LNG did not alter myeloid DC (mDC) viability (Fig. S1), but that LNG concentrations > 0.250 μM inhibited expression of CD80 and CD86 by mDCs responding to poly I:C stimulation (Fig. 1a–c). Even more robust was LNG-mediated suppression of CD40 expression (Fig. 1d). This latter result was congruent with inhibition of CD40 expression in MPA-treated human DCs stimulated *in vitro* with poly I:C^16^, and reduced CD40 expression in DCs isolated from the draining lymph nodes (DLNs) of MPA-treated mice 2 days after corneal HSV-1 infection^14^.

**Figure 2:**
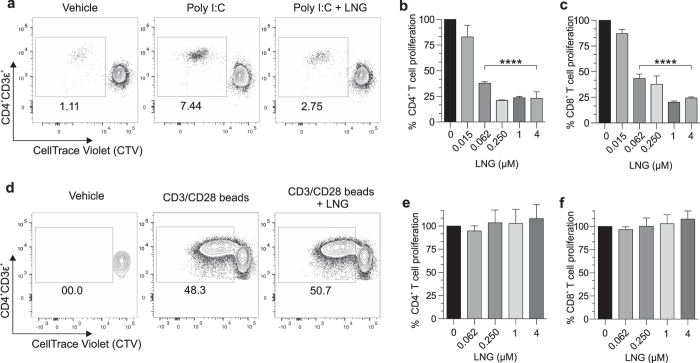
LNG inhibits human DC function. (**a–c**) Negatively selected human DCs were LNG-treated and poly I:C stimulated as described in Fig. 1, then co-cultured with CTV-labeled naïve allogeneic T cells. Co-cultures were maintained 7 days, then T cells immunostained for flow cytometric analysis of proliferation. (**a**) Representative contour plots of CD4^+^ T cell proliferation from co-cultures with untreated or LNG (4 μM)-treated DCs (numbers denote percentages of proliferating CD3ε^+^CD4^+^ T cells). (**b–c**) Proliferation of (**b**) CD4^+^ and (**c**) CD8^+^ T cells from co-cultures with untreated or LNG-treated DCs (data from 6 independent experiments normalized and analyzed as detailed in Materials and Methods); (bars indicate means ± SD). (**d–f**) CTV-labeled naïve allogeneic T cells were incubated with LNG or vehicle overnight, then stimulated with beads coated with anti-CD3 and anti-CD28 antibodies. T cell cultures were maintained 5 days, and cells immunostained for flow cytometric analysis of proliferation. (**d**) Representative contour plots of T cell proliferation (4 μM LNG); (numbers identify percentages of proliferating CD3ε^+^CD4^+^ T cells). Proliferation of (**e**) CD4^+^ and (**f**) CD8^+^ T cells in T cell-only cultures treated with vehicle or LNG (data from 6 independent experiments that were normalized and analyzed as defined in Materials and Methods); (bars denote means ± SD). All statistical analyses were performed using 1-way ANOVA with Dunnett’s multiple comparisons test, ****p < 0.0001.

**Figure 3:**
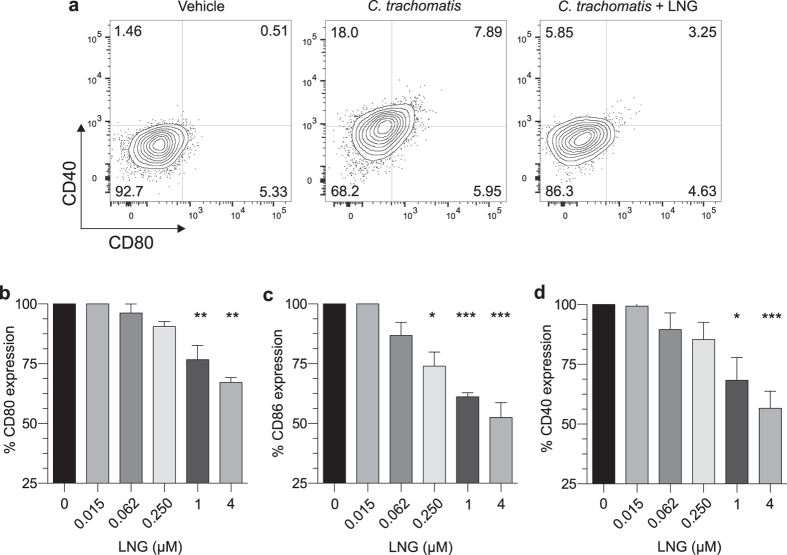
LNG inhibits human DC response to *C. trachomatis* stimulation. Negatively selected human DCs were incubated for 24 h with indicated LNG concentrations or vehicle, then incubated for 24 h with inactivated *C. trachomatis* (MOI = 0.1 prior to inactivation). To identify live DCs, cells were stained as described in Fig. 1. (**a**) Representative contour plots displaying CD40 and CD80 expression by untreated and LNG (4 μM)-treated mDCs stimulated with *C. trachomatis*; quadrant numbers indicate percent expression. (**b–d**) mDC expression of (**b**) CD80, (**c**) CD86, and (**d**) CD40 induced by *C. trachomatis*. Data from 8 independent experiments were normalized as described in Materials and Methods (i.e., by designating vehicle-only cultures as 100% proliferation); (bars denote means ± SD). Statistical analyses made using 1-way ANOVA with Dunnett’s multiple comparisons test, *p < 0.05; **p < 0.01; and ***p < 0.001.

Because LNG suppressed human DC activation *in vitro*, we posited it also inhibits DC function. To test this hypothesis, negatively selected human DCs from 6 individuals were sequentially administered vehicle alone or LNG for 24 h; poly I:C stimulated for 24 h; and incubated for 7 days with fluorescently-labeled naïve allogeneic T cells. Using flow cytometry, we detected significantly less CD4^+^ and CD8^+^ T cell proliferation in co-cultures that contained LNG-treated DCs (Fig. 2a–c). Of note, all but the lowest selected LNG concentration significantly inhibited this proliferation. Conversely, the stimulation of T cell-only cultures with anti-CD3 and anti-CD28 antibodies showed LNG treatment had no direct effects on T cell capacity to proliferate (Fig. 2d–f). Extending these results beyond DC response to TLR3 agonist stimulation, we also found that CD40 expression was significantly blunted when LNG-treated human DCs were stimulated *in vitro* with inactivated *C. trachomatis* (Fig. 3). Of note, stimulation with poly I:C or *C. trachomatis* produced a markedly different pattern of co-stimulatory molecule expression (Figs 1a and 3a), indicating that different DC activation pathways had been triggered by stimulation with this particular TLR3 agonist or Gram-negative bacterium.

### LNG suppressed murine host DC response to *C. trachomatis* infection

**Figure 4:**
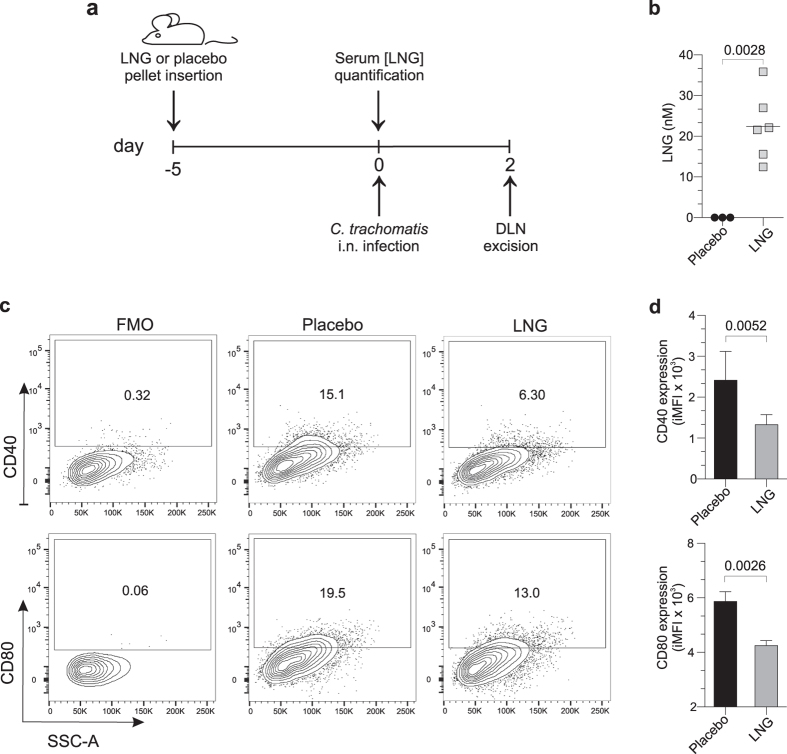
LNG reduces DC activation elicited by *Chlamydia* infection. (**a**) LNG or matching placebo pellets were implanted into Balb/cJ female mice, and peripheral blood collected 5 days later to measure serum LNG concentrations. (**b**) Serum LNG levels; symbols represent values from individual mice and horizontal lines indicate means. (**c–d**) Other mice implanted with pellets as described in (**a**) were i.n. infected with 10^5^ IFU of live *C. trachomatis*. Mice were euthanized at 2 dpi, and DLNs collected to assess DC activation by flow cytometry. Representative contour plots show (**c**) CD40 and CD80 expression in live CD11c^hi^MHC-II^+^CD8α^-^CD11b^+^ cells (mDCs); numbers denote percent expression. (**d**) mDC expression of CD40 and CD80 from placebo- or LNG-pelleted mice from two independent experiments with 6 mice per condition; bars designate mean values ± S.D. Statistical analyses performed with 2-tailed unpaired Student *t* tests.

Since LNG inhibited *in vitro* DC activation, we hypothesized that antecedent treatment of mice with LNG also reduces activation of DCs elicited by *C. trachomatis* infection. As an important experimental consideration, antecedent progestin treatment is exploited in numerous animal models of genital infection (including murine *Chlamydia* models that use intravaginal, trans-cervical, and direct ovarian bursal inoculation) to produce uniform susceptibility to infection^8^,^19^,^20^,^21^. We thus tested our hypothesis in a *C. trachomatis* respiratory infection model, a model in which uniform infection susceptibility is achieved without antecedent progestin treatment. In other words, the use of a non-genital infection route was the best way to prevent identification of between-group differences in DC function or T cell expansion spuriously created by a differential susceptibility of untreated and LNG-treated mice to genital infection. Using the respiratory model of infection, wild type Balb/cJ female mice were subcutaneously implanted with 21-d sustained release pellets containing 50 mg LNG or matching placebo pellet (Fig. 4a). With initial studies in this model, we collected blood 5 d after pellet insertion to quantify serum LNG concentrations (Fig. 4b). To assess LNG effects on DC activation, other mice were intranasally (i.n.) infected with *C. trachomatis* 5 days after LNG- or placebo-pellet insertion. Of note, we utilized live (not inactivated) *Chlamydia* in our exploration of this respiratory infection model, as only the former generated a mature DC phenotype that promoted pathogen-specific protective immunity^22^,^23^. Controls and LNG-pelleted mice were euthanized at 2 days post infection (dpi), and DLNs were processed for flow cytometric analysis of DC activation. Consistent with our human DC data, we found that antecedent LNG treatment of mice significantly reduced DC expression of CD40 and CD80 induced by *C. trachomatis* infection (Fig. 4c,d).

### LNG dampened murine host T cell response to *C. trachomatis* infection

**Figure 5:**
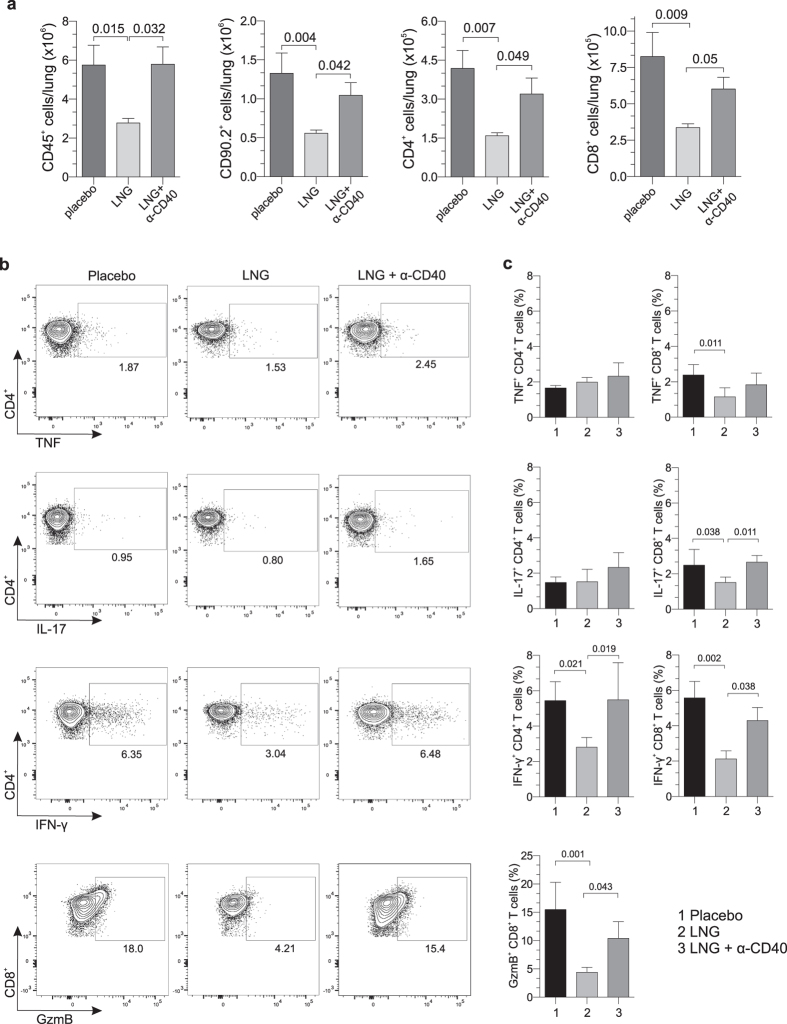
LNG impairs T cell expansion and pathogen-specific T cell effector function. Mice were administered LNG or matching placebo pellet 5 days before i.n infection with 10^5^ IFU of live *C. trachomatis*. Other LNG-pelleted mice received agonist anti-CD40 mAb injection 1 day after infection. (**a**) Mice were euthanized at 12 dpi, and lungs processed into single-cell suspensions to enumerate CD45^+^, CD90.2^+^, CD4^+^, and CD8^+^ cells by flow cytometry. (**b-c**) In separate studies, groups of mice were treated and infected identically as described in (**a**). Mice were euthanized at 12 dpi, and lungs processed into single-cell suspensions to quantify GrzB levels inside CD8^+^ T cells and define *Chlamydia*-specific CD4^+^ and CD8^+^ T cell effector function. For the latter, BMDCs were obtained from untreated, uninfected syngeneic mice as defined in Materials and Methods, and stimulated overnight with *C. trachomatis* (MOI = 0.1). The next day, *Chlamydia*-infected mice (i.e., at 12 dpi) were euthanized, and single-cell suspensions of lung tissue incubated 24 h with the *Chlamydia*-activated BMDCs to quantify T cell secretion of IFN-γ, TNF, and IL-17 by flow cytometry. (**b**) Representative contour plots display production of IFN-γ, TNF, and IL-17 by CD4^+^ T cells and GzmB levels inside CD8^+^ T cells. (**c**) Between group-differences in levels of GzmB (CD8^+^ T cells) or intracellular accumulation of IFN-γ, TNF, and IL-17 (CD4^+^ and CD8^+^ T cells) (bars indicate mean values ± S.D). Data in (**a**) and (**c**) are from 2 independent experiments with 6 mice per group. Statistical analyses were completed using 1-way ANOVA with Dunnett’s multiple comparisons test.

In separate experiments, mice received LNG or matching placebo pellet 5 days before infection, while other LNG-pelleted mice received agonist anti-CD40 monoclonal antibody 1 day after infection. Animals from all 3 groups were euthanized at 12 dpi, and flow cytometric analysis of lung tissue showed there were significantly fewer CD4^+^ and CD8^+^ T cells in mice receiving LNG pellet alone (Fig. 5a). LNG treatment was also associated with significantly less interferon (IFN)-γ secretion when lung-resident CD4^+^ and CD8^+^ T cells were stimulated *ex vivo* with *Chlamydia*-infected bone marrow-derived DCs (BMDCs) and significantly reduced CD8^+^ T cell levels of the serine protease granzyme B (GzmB) (Fig. 5b,c). Highlighting the importance of CD40-CD154 interactions for both T cell expansion and development of T cell effector function in this *Chlamydia* respiratory infection model, we conversely found comparable T cell numbers and T cell levels of IFN-γ and GzmB among LNG-pelleted mice administered agonist anti-CD40 antibody and mice treated with placebo pellet (Fig. 5a and c).

### LNG impaired clearance of intranasal *C. trachomatis* infection

**Figure 6:**
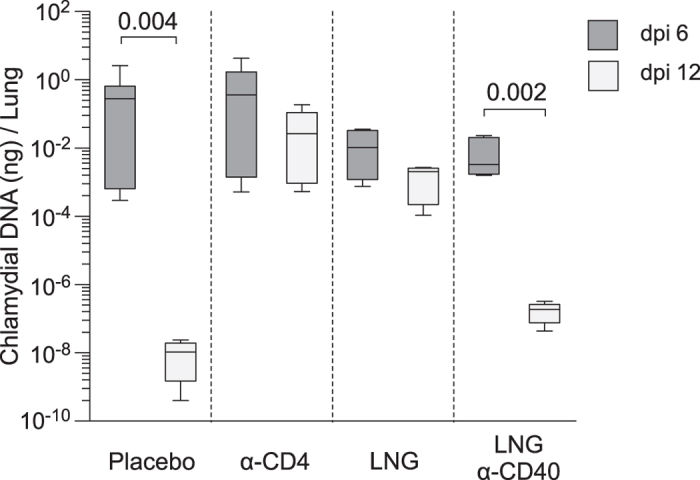
LNG diminishes *C. trachomatis* clearance from pulmonary tissue. Groups of mice were administered LNG or matching pellets 5 d prior to i.n infection with 10^5^ IFU of live *C. trachomatis*. Other groups of LNG-pelleted mice received agonist anti-CD40 mAb 1 day after chlamydial infection or CD4^+^ cell-depleting mAb 1 d prior and every other day for study duration. Animals were euthanized at 6 dpi or 12 dpi, and both lung lobes excised to quantify chlamydial DNA by RT-qPCR; (bars indicate mean ± C.I); (data from 2 independent experiments with 6 animals per group). *Chlamydia* levels at each dpi were compared using the 2-tailed unpaired Student *t* test.

**Figure 7:**
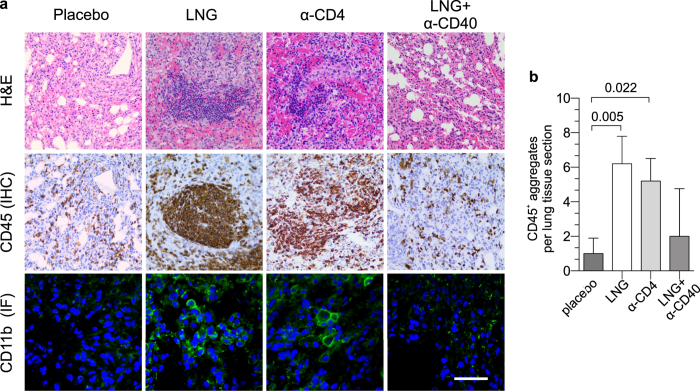
Greater chlamydial burden in LNG-treated mice elicits increased mononuclear cell inflammation. In separate studies, groups of mice were treated and infected identically as described in Fig. 6. Mice were euthanized at 12 dpi, and pulmonary tissue processed to define inflammation by histology. (**a**) Representative results from hematoxylin and eosin, IHC, and IF staining depicts the increased mononuclear cell inflammation seen in the lungs of LNG-treated- and CD4^+^ cell-depleted mice; IF staining: CD11b^+^ (green), DAPI (blue); black and white bars denote 200 μm and 20 μm, respectively. (**b**) Quantification of pulmonary CD45^+^ inflammatory aggregates identified by IHC staining (5 high-power fields examined per section); (bars denote mean ± S.D.). Data are from 2 independent experiments with 6 mice per group. Statistical analyses were performed using 1-way ANOVA with Dunnett’s multiple comparisons test.

As previously established for mice, T_H_1 immunity is critical for control of *C. trachomatis* infection^24^. Upon observing diminished T cell expansion and T_H_1 effector function in LNG-treated mice infected with *C. trachomatis*, we posited that eradication of pulmonary infection is also impaired by LNG treatment. To explore this possibility, groups of mice received LNG or matching placebo pellet 5 days before infection. Other LNG-pelleted mice received agonist anti-CD40 antibody 1 day after infection, while other placebo-pelleted mice received CD4^+^ cell-depleting mAb 1 day prior to infection and for the duration of the study. Animals from each group were euthanized at 6 dpi or 12 dpi, and consistent with prior reports^24^,^25^,^26^, *Chlamydia* was effectively cleared from the lungs of placebo-pelleted mice between 6 and 12 dpi. In comparison, CD4^+^ T cell-depleted mice less successfully controlled infection (Fig. 6). Remarkably, clearance was similarly impaired in LNG-pelleted mice, whereas clearance in LNG-pelleted mice administered agonist anti-CD40 antibody was restored to levels detected in placebo-pelleted controls (Fig. 6). Congruent with delayed clearance in LNG-treated mice, we saw significantly more myeloid (CD45^+^CD11b^+^) mononuclear cell aggregates in the lungs of LNG-treated mice at 12 dpi compared to placebo-pelleted controls (Fig. 7). CD4^+^ cell depletion caused comparable increase in these myeloid cell aggregates, whereas treatment of LNG-pelleted mice with agonist anti-CD40 antibody reduced the inflammatory response to the levels seen in placebo-pelleted controls (Fig. 7). These studies thus revealed LNG treatment of mice prior to intranasal *C. trachomatis* infection delays both pathogen clearance and resolution of pulmonary inflammation.

## Discussion

A 2015 publication provided interesting first indication that LNG influences pathogen clearance. Among the 66 women in that study diagnosed with high-risk HPV genital infection prior to IUS insertion, 70% initiating copper-containing IUS use vs. 42% initiating LNG-IUS use cleared infection during the first year after insertion^12^. Such results implied LNG, and not the mere presence of an IUS, was the variable more closely associated with delayed HPV clearance. Of note, that study did not comparably assess HPV clearance in a group of women not using hormonal contraception. Moreover, while T cell responses are considered important for HPV eradication^27^, that study was not designed to determine if LNG-mediated effects on T cell immunity contributed to the greater persistence of HPV among LNG-IUS users.

Herein, we sought to define the impact of LNG on early adaptive immunity. In initial exploration of the *in vitro* effects of LNG on human DC activation and function, we saw LNG reduce CD40 expression in DCs responding to poly I:C and reduce DC capacity to induce T cell proliferation. The former observation likely predicted the latter, as CD40 signaling elicits changes that make DCs more effective promoters of T cell activation^28^,^29^,^30^,^31^. Remarkably, impaired DC capacity to promote T cell proliferation in our study was produced with LNG concentrations as low as 0.062 μM. Although peak and steady-state serum concentrations of LNG after LNG-IUS insertion are typically less than 1.2 nM^32^,^33^,^34^,^35^, systemic levels do not reflect the concentrations achieved in female genital mucosal tissue. Endometrial LNG tissue levels measured 1–2 months after LNG-IUS insertion ranged between 174 ng–660 ng^36^, and we approximated these tissue levels create LNG concentrations between 2.8 μM–10.6 μM in the typical 200 μl volume of uterine fluid^37^,^38^. These values indicate the LNG-mediated inhibition of human DC response to TLR3 agonist stimulation observed in our study occurred at pharmacologically relevant levels. Comparable levels of LNG likewise impaired *in vitro* activation of human DCs responding to *C. trachomatis*. Our results thus reveal LNG blunts human DC response to an immunostimulant used to mimic viral infection and a bacterial pathogen, and are congruent with studies reporting reduced *in vitro* activation and function of progesterone-treated mouse DCs and MPA-treated human DCs^16^,^39^. Though it was earlier postulated that greater PR specificity makes LNG less likely than MPA to impair DC function^40^, our work also newly indicates that suppression of DC activation and function are immunomodulatory properties shared by MPA and LNG.

Since LNG reduced CD40 expression in DCs responding to *in vitro C. trachomatis* stimulation, we explored effects of LNG on DC activation, T cell expansion, and T cell effector function in a murine model of *Chlamydia* respiratory infection. This model was chosen in general because CD40-CD154 interactions promote T_H_1-type immune responses needed to combat intracellular pathogens^41^,^42^, and specifically because CD40 knockout mice displayed prolonged infection courses after chlamydial infection^43^. Congruent with the reduced CD40 expression in LNG-treated human DCs, we saw significantly lower CD40 expression in DCs isolated from DLNs of LNG-treated mice at 2 dpi. Signifying this degree of inhibition of DC activation was a biologically relevant response, the impaired T cell expansion in lungs of LNG-treated mice was abrogated when LNG-treated mice also received agonist anti-CD40 antibody. Using this mouse model, we further showed that LNG impairs development of *Chlamydia*-specific T_H_1-type effector function. The latter finding result is compatible with prior studies reporting that DC CD40 expression is critical for *Mycobacterium tuberculosis*-specific T cell expansion^44^,^45^ and that CD40-CD154 interactions optimize formation of pathogen-specific T cell effector function^46^,^47^,^48^.

Consistent with impaired development of *Chlamydia*-specific T_H_1 immunity in LNG-treated mice was its association with delayed clearance of pulmonary infection. Remarkably, chlamydial load in LNG-treated mice was increased similarly to levels seen in mice depleted of CD4^+^ T cells. Moreover, delayed chlamydial clearance slowed resolution of the myeloid mononuclear cell-dominated pulmonary infiltrate. Conversely, administration of agonist anti-CD40 mAb to LNG-treated mice at dpi 1 produced *Chlamydia* burden and pulmonary inflammation at 12 dpi that closely resembled that seen in placebo-pelleted controls. Thus, while our use of sustained-release LNG pellets in a mouse model of respiratory *C. trachomatis* infection may not fully recapitulate the immunomodulatory effects of LNG-IUS use in the genital tract of women, the elevated chlamydial burden we detected in LNG-treated mice at 12 dpi is entirely consistent with the delayed eradication of genital HPV observed in women using LNG-IUS^12^.

In summary, this study newly identifies mechanisms by which LNG suppresses early adaptive immune responses and inhibits host ability to eradicate an intracellular bacterial pathogen from mucosal tissue. These findings may have specific clinical relevance, as any variable that delays pathogen clearance increases the chance for pathogen transmission from infected to uninfected individuals. Also of potential clinical relevance is the more exuberant inflammation found in the lungs of LNG-treated mice, as the inflammatory response elicited in women during a chronic genital *C. trachomatis* was identified as a risk factor for development of PID and tubal-factor infertility^49^,^50^. Our results thus ultimately serve to highlight the need for clinical studies that define formation development of pathogen-specific immune responses and rates of pathogen clearance among women using LNG-IUSs.

## Methods

### *In vitro* procedures

Buffy coats from de-identified individuals were provided by the Central-Southeast Ohio Region American Red Cross. Peripheral blood mononuclear cells (PBMCs) were isolated from these buffy coats by density gradient centrifugation (Ficoll-Paque^TM^ PLUS) (Healthcare Bio-Sciences AB, Uppsala, Sweden), and cryopreserved in 10% dimethyl sulfoxide (DMSO) (Mediatech, Manassas, VA, USA) and 90% fetal bovine serum (FBS) (Atlanta Biologicals, Flowery Branch, GA, USA). Thawed PBMCs were used to isolate primary DCs by negative immunomagnetic selection (EasySep™ Human Pan-DC Pre-Enrichment Kit) (StemCell Technologies, Vancouver, Canada). As indicated, allogeneic PBMC were labeled with CellTrace™ Violet Cell Proliferation Dye (CTV) (Invitrogen, Eugene, OR, USA), and naïve T cells were isolated using the Pan Naïve T Cell Isolation Kit (Miltenyi Biotec, San Diego, CA, USA). LNG (Sigma-Aldrich, St. Louis, MO) solubilized in DMSO was used to prepare 100 μM stock solutions. *C. trachomatis* serovar L2 (strain 434; ATCC^®^ VR-902B) was grown on McCoy cells (ATCC^®^ CRL-1696), and elementary bodies (EB) isolated and stored at −80 °C in sucrose-phosphate-glutamate buffer (SPG)^51^. Prior to experimental use, *C. trachomatis* inclusion-forming units (IFU) were enumerated as previously described^52^.

To assess DC activation, human primary DCs re-suspended in *X-VIVO* 20 (Lonza, Walkersville, MD, USA) containing 10% AB human serum (Atlanta Biologicals) were placed in individuals wells of 96-well, round bottom polypropylene plates (Corning Inc., New York, NY, USA) (5 × 10^4^ DC/well). Cells were incubated 12 h at 37 °C in a 5% CO_2_ atmosphere using media + select LNG concentrations (final DMSO concentrations in untreated and LNG-treated wells were <0.001%). Cultures were administered vehicle or poly I:C (1.5 μg/mL) (InvivoGen, San Diego, CA, USA), and incubated an additional 24 h. Other vehicle-or LNG-treated DCs were stimulated with *C. trachomatis* (MOI = 0.1) previously inactivated by incubation at 56 °C for 15 min. DCs were harvested to evaluate co-stimulatory molecule expression by flow cytometry. To assess DC function, other negatively selected human DCs were sequentially plated (2.5 × 10^3^ DC/well); vehicle- or LNG-treated; and poly I:C stimulated. Allogeneic, CTV-labeled naïve T cells (1 × 10^5^ cells/well) were co-cultured with DCs for 7 d at 37 °C in 5% CO_2_ (with media replenished every third day), and T cell proliferation measured by flow cytometry. To evaluate the direct effects of LNG on T cell proliferation, CTV-labeled T cells (10^5^ cells/well) were incubated for 12 h with vehicle or indicated LNG concentrations. Cells were stimulated with Dynabeads^®^ Human T-Activator CD3/CD28 (Life Technologies, Oslo, Norway) (1:8 bead: cell ratio), and incubated 5 d (media was not replenished in this assay). T cell proliferation was assessed by flow cytometry.

### *In vivo* and *ex vivo* procedures

Mouse studies were approved by The OSU Institutional Animal Care and Use Committee and performed in accordance with institutional welfare guidelines. For pellet insertion, 6- to 8-week-old wild type female Balb/cJ mice (The Jackson Laboratory, Bar Harbor, ME) were anesthetized by intraperitoneal (i.p.) injection of 0.18 mg xylazine (Lloyd Laboratories, Shenandoah IA, USA) and 1.8 mg ketamine hydrochloride (JHP Pharmaceuticals, LLC Rochester MI, USA). Sedated mice were surgically implanted^14^ with 21-d sustained release pellets containing 50 mg LNG or matching placebo pellets (Innovative Research of America, Sarasota, FL, USA). As indicated, mice were euthanized 5 d after pellet insertion to measure serum LNG levels via RIA according to manufacturer’s instructions (Immunometrics, London, U.K.). Other LNG- or placebo-pelleted mice were anesthetized for intranasal (i.n.) *Chlamydia trachomatis* infection (10^5^ IFU). Where indicated, beginning 1 d before infection, mice received 100 μg i.p. injection of an anti-CD4 mAb (GK 1.5) (BioXCell, Lebanon, NH) every 48 h until study termination. Efficiency of CD4^+^ T cell depletion was evaluated in peripheral blood and lung tissue by flow cytometry (Fig. S3), and was routinely >97%. Also where indicated, LNG-pelleted mice were intravenously (i.v.) administered 100 μg agonist anti-CD40 mAb (FGK 4.5) (Bio X cell, Lebanon, USA).

To assess DC activation, mice were euthanized at 2 dpi, and cervical lymph nodes (i.e., DLNs) excised, digested with collagenase D (Roche, Indianapolis, IN, USA) and deoxyribonuclease I (Sigma-Aldrich) for 1 h at 37 °C, and processed into single-cell suspension for flow cytometric analysis. To assess T cell expansion elicited in the lungs after i.n. *C. trachomatis* infection, mice were euthanized at 12 dpi. Lungs were excised and processed for flow cytometry by incubation for 1 h at 37 °C in RPMI-1640 supplemented with 10% FBS, 2 mM L-glutamine, 1 mM sodium pyruvate, non-essential amino acids, 50 μM 2-ME, 100 U/mL penicillin, 100 μg/mL streptomycin, and 50 μg/mL gentamycin (Mediatech) (hereafter termed complete media), 1 mg/mL collagenase D, and 0.25 mg/mL deoxyribonuclease I. As previously described^14^, GzmB levels in T cells isolated from the lungs of uninfected (Fig. S2) and *Chlamydia*-infected mice were evaluated by flow cytometry. To define the effects of LNG on *Chlamydia*-specific T cell effector function, BMDCs were generated using uninfected female Balb/cJ mouse. As described elsewhere^53^,^54^, BMDCs were incubated in complete media at 37 °C with recombinant murine GM-CSF for 3 d, then GM-CSF and recombinant murine IL-4 for 3 d (R&D Systems, Inc., Minneapolis, MN). BMDCs were stimulated with live *C. trachomatis* (MOI = 0.1) for 24 h, and mixed at a 1:1 ratio with single-cell suspensions from the lungs of uninfected and *Chlamydia*-infected mice. Co-cultures were incubated for 18 h, and GolgiPlug™ (BD Biosciences) added during the last 6 h. As previously described^14^, T cells were fixed and permeabilized to quantify the intracellular accumulation of IFN-γ, TNF, and IL-17 using flow cytometry. To measure effects of LNG on chlamydial clearance from the lungs, mice were euthanized at 6 and 12 dpi. Lung lobes were excised, and total DNA extracted using Genomic DNA buffer sets with Genomic-tip 100/G kits following manufacturer’s instructions (Qiagen, Hilden, Germany). DNA was quantified (260/280 and 260/230 ratios were >1.8). *C. trachomatis* DNA obtained from purified EBs with DNeasy Blood & Tissue kits (Qiagen), was used to generate standard curves that defined *C. trachomatis* DNA levels in infected lungs^55^. Where indicated, mice were euthanized at 12 dpi, and excised lungs fixed with 4% methanol-free formaldehyde (Thermo Scientific, Rockford, IL, USA). After 24 h, samples were paraffin embedded, sectioned, and hematoxylin and eosin stained. Where noted, paraffin-embedded sections underwent immunohistochemical (IHC) staining to identify CD45^+^ (clone 30-F11, BD Biosciences) cells. For immunofluorescence (IF) assays, 10 μm sections from paraffin-embedded lungs were mounted on glass slides, and de-paraffinized by sequential immersion in 100% xylene, 100% ethanol, 96% ethanol, and sterile DEPC-treated water. Antigen retrieval was performed using 10 mM sodium citrate buffer (pH 6.0) containing 0.05% Tween 20 (Sigma-Aldrich) (20 minutes at 95 °C). Treated sections were washed in PBS, and incubated for 12 h at 4 °C with 10% normal donkey serum; 1 h at ambient temperate with rabbit anti-CD11b (clone EPR1344); and 1 h with Alexa Fluor^®^ 488-conjugated donkey anti-rabbit IgG (Abcam, Cambridge MA, USA) (antibodies were diluted in PBS supplemented with 1% BSA and 0.05% Tween 20). Sections were counterstained with DAPI, and inflammatory infiltrates evaluated using the Olympus FV1000 spectral confocal microscope (Tokyo, Japan).

### Flow cytometry

Where indicated, cells were first stained with Live/Dead Fixable near-IR (Invitrogen, Eugene, OR, USA). Human cells were stained with anti-HLA-DR FITC (G46-6), anti-CD11c PE (B-ly6), anti-CD80 PE-Cy7 (L307.4), anti-CD40 APC (5C3), anti-CD86 BV510 (FUN-1), anti-CD123 BV421 (9F5), CD3 FITC (UCHT1), anti-CD4 PE (RPA-T4), or CD8 PE-Cy7 (RPA-T8) (all BD Biosciences). Mouse cells were stained with anti-IL-17 A PE (TC11-18H10), anti-CD8a V450 (53-6.7) anti-CD40 FITC (3/23) anti-CD80 APC (16–10A1), anti-CD11b BV510 (M1/70) (all BD Biosciences); anti- IFN-γ APC (XM61.2), anti-CD11b PE-Cy7 (M1/70), anti-CD4 PE-Cy7 (RM4–5), anti-CD4 PE (RM4–4) was used when anti-CD4^+^ mAb was used to deplete CD4^+^ cell, anti-CD45 PerCP (30-F11), anti-CD90.2 PerCP (30-H12) (all BioLegend San Diego, CA); anti-TNF FITC (MP6-XT22), anti-CD90.2 FITC (54-2.1) anti-Granzyme B PE (NGZB), anti-B220 APC (RA3-6BC), anti-CD11c PE-Cy7 (N418), anti-CD90.2 PerCP-eF710 (30-H12), anti-CD11c PE (N418), or anti-MHC-II PE-Cy7 (M5/114.15.2) (all eBioscience, San Diego, CA). Samples were run on a FACSCanto II (BD Biosciences, San Jose, CA, USA), and analyzed using FACS Diva (BD Biosciences) and FlowJo software (Tree Star Inc., Ashland OR). Fluorescence minus one controls were used to define costimulatory molecule expression by DCs stimulated with poly I:C or chlamydial antigen.

### Statistical considerations

Statistical analyses were performed using Prism 6 software (GraphPad, La Jolla, CA, USA). LNG-mediated effects on DC activation were defined by comparing percentages of activated DCs in vehicle-treated vs. LNG-treated cultures (data normalized by designating vehicle-only cultures as 100% activation); (T cell proliferation values were similarly normalized designating proliferation in vehicle-only controls as 100%). Normal distribution was tested by D’Agostino & Pearson omnibus test or evaluation of the residuals (when experimental sample numbers were <8). Differences among paired samples between 2 groups were compared by unpaired Student *t* or Mann–Whitney U tests (depending on data distribution). For multiple comparisons, 1-way ANOVA with Dunnett’s or Tukey’s post hoc test (parametric distribution) or Friedman test with Dunn’s or Kruskal-Wallis post hoc test (nonparametric distribution) were used. For all analyses, *P* values < 0.05 were designated as statistically significant.

## Additional Information

**How to cite this article**: Quispe Calla, N. E. *et al*. Dendritic cell function and pathogen-specific T cell immunity are inhibited in mice administered levonorgestrel prior to intranasal *Chlamydia trachomatis* infection. *Sci. Rep.* **6**, 37723; doi: 10.1038/srep37723 (2016).

**Publisher's note:** Springer Nature remains neutral with regard to jurisdictional claims in published maps and institutional affiliations.

## Supplementary Material

Supplementary Material
