# Supplementary material for: Dendritic cell function and pathogen-specific T cell immunity are inhibited in mice administered levonorgestrel prior to intranasal Chlamydia trachomatis infection
Source: Sci Rep. 2016 Nov 28;6:37723. doi: 10.1038/srep37723 (PMC5125275; doi:10.1038/srep37723)
Supplement: Supplementary Material [file srep37723-s1.pdf]

Supplementary material

Dendritic cell function and pathogen-specific T cell immunity are inhibited in mice administered levonorgestrel prior to intranasal *Chlamydia trachomatis* infection

Nirk E Quispe Calla[1], Rodolfo D Vicetti Miguel[1], Ao Mei[1], Shumin Fan[1], Jocelyn R Gilmore[1], Thomas L Cherpes[1, 2]

[1]Department of Microbial infection & Immunity, [2]Department of Obstetrics & Gynecology

The Ohio State University College of Medicine, Columbus, OH, 43210, USA

Corresponding authors:

Nirk E. Quispe Calla, MD

Biomedical Research Tower, Room 740,460 West 12th Ave., Columbus, OH 43210, USA

Email address: quispecalla.1@osu.edu Telephone: 614.688.2165 Fax: 614.292.9616

Rodolfo D. Vicetti Miguel, MD

Biomedical Research Tower, Room 731, 460 West 12th Ave., Columbus, OH 43210, USA

Email address: vicettimiguel.1@osu.edu Telephone: 614.688.1862 Fax: 614.292.9616

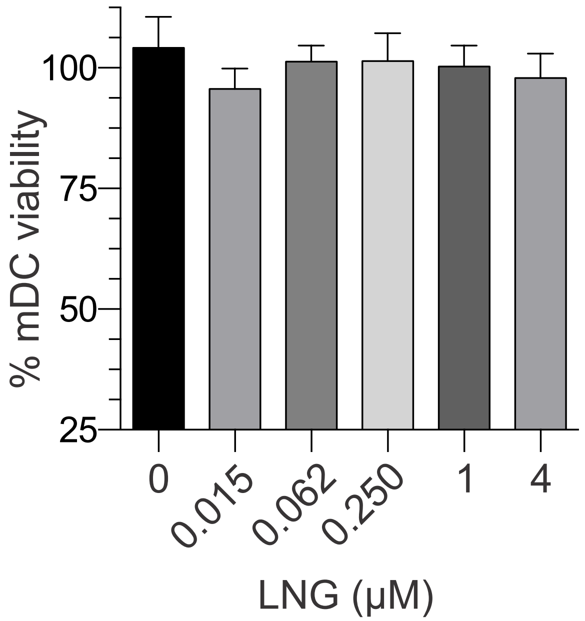

Figure S1. **LNG does not affect mDC viability**. Negatively selected human primary mDCs were incubated for 24h with select LNG concentrations or vehicle only. Poly I:C (1.5μg/mL) was added, and DCs incubated an additional 24h. Cells were harvested, stained with Live/Dead near-IR dye, and immunostained to identify mDC (HLA-DR^{+}CD11c^{+}CD123^{-}) populations. mDC viability was normalized by defining the viability in cells treated with vehicle and poly I:C as 100%. Bars indicate mean ± SD from 8 independent experiments. Comparisons were made using 1-way ANOVA.

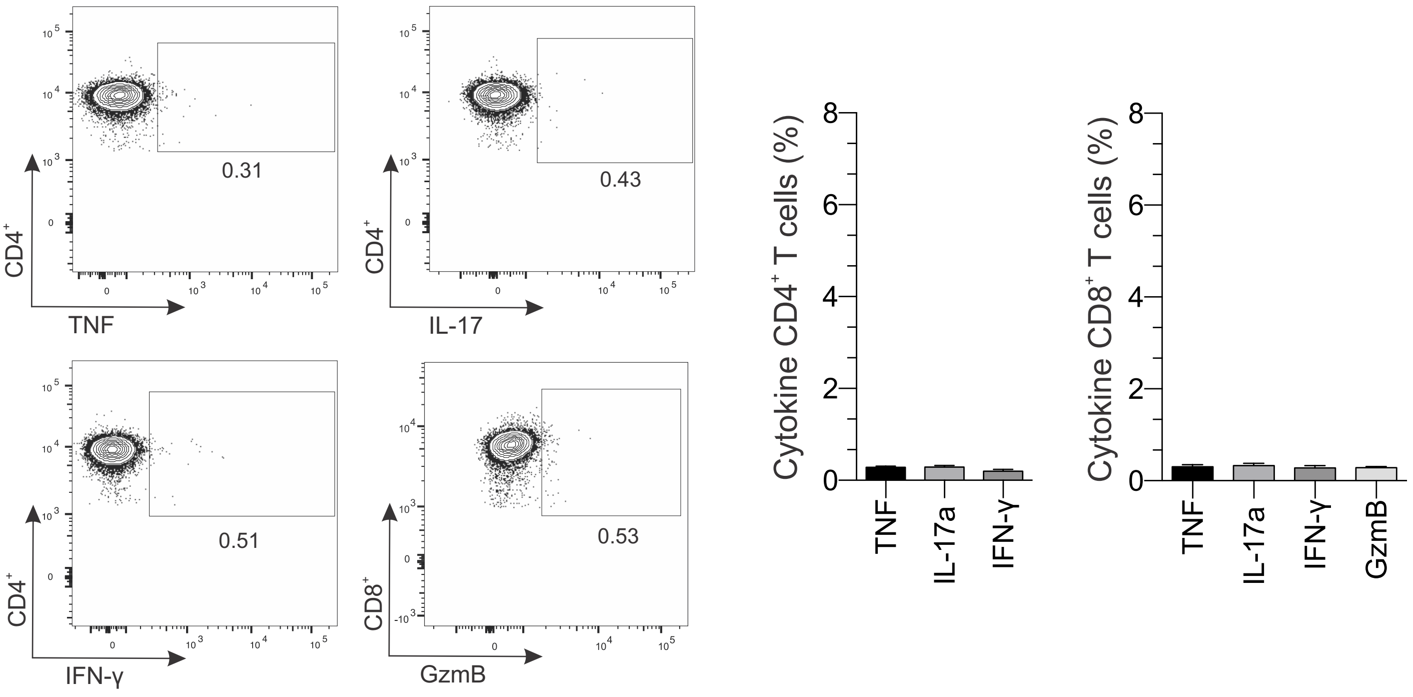

Figure S2. ***Chlamydia*-specific T cell effector function in uninfected, untreated mice**. BMDCs were generated from untreated, uninfected syngeneic mice and stimulated with *C. trachomatis* (MOI = 0.1). Other uninfected, untreated mice were euthanized and their lungs processed into single-cell suspension. T cells from these suspensions were immediately immunostained to quantify intracellular GzmB levels or co-cultured for 18h with the *Chlamydia*-activated BMDCs to assess intracellular accumulation of IFN-γ, TNF and IL-17 by flow cytometry. Left panels show representative contour plots of GzmB, IFN-γ, TNF, and IL-17 levels in $CD4^+$ and $CD8^+$ T cells. Right panels are histograms quantifying cytokine production by $CD4^+$ and $CD8^+$ T cells. Bars depict mean ±S.D. from 2 independent experiments (comparisons were made using 1-way ANOVA).

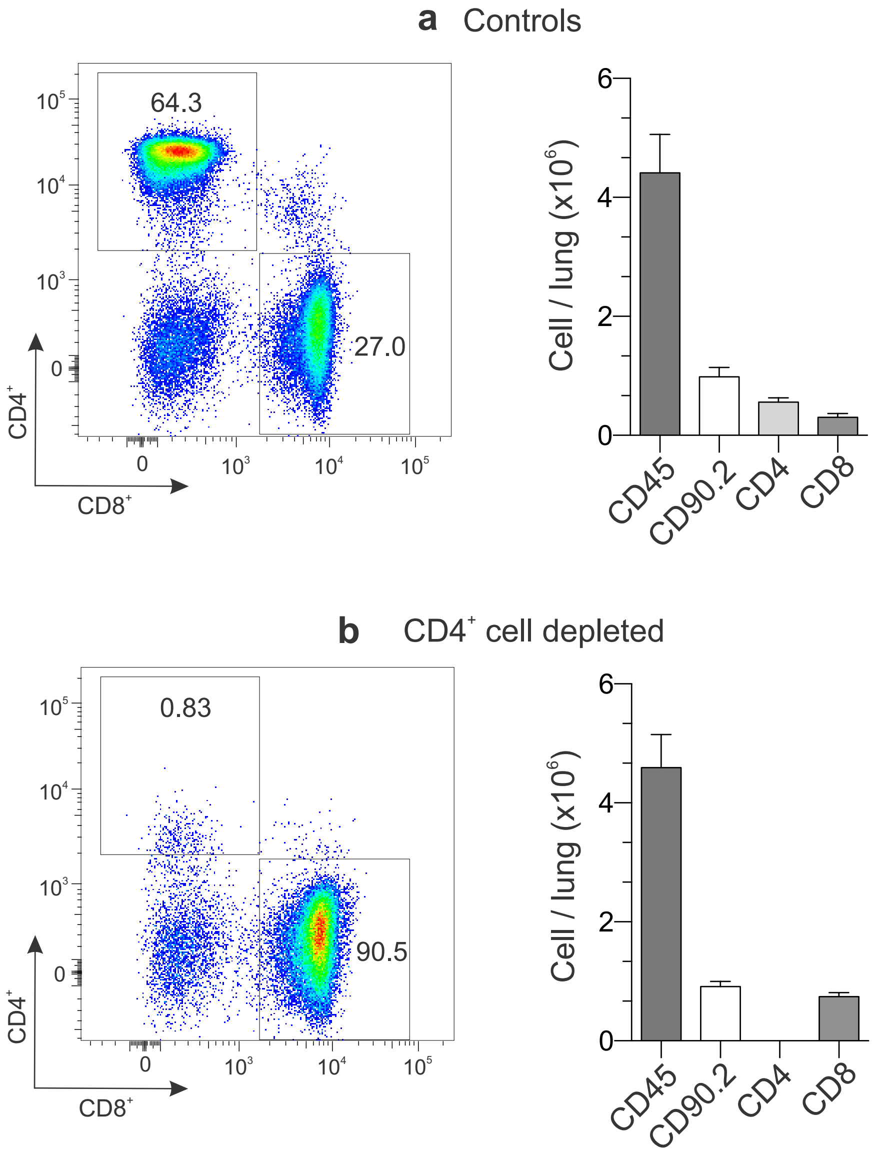

Figure S3. Efficacy of $CD4^+$ T cell depletion from the lungs of *Chlamydia*-infected mice by anti-CD4 mAb administration. One day prior to infection, and every other day for study duration, mice were administered (a) isotype control Ab or (b) anti-CD4 mAb. Animals were euthanized at 12 dpi and lungs excised, processed into single-cell suspension and immunostained to quantify leukocytes as detailed in Material and Methods. Left panels show representative contour plots of $CD4^+$ and $CD8^+$ T cells (numbers in quadrants denote population percentages); right are histograms that enumerate leukocyte subpopulations. Bars indicate mean ±S.D. from 2 independent experiments.
